# Value of gait analysis for measuring disease severity using inertial sensors in patients with multiple sclerosis: protocol for a systematic review and meta-analysis

**DOI:** 10.1186/s13643-018-0918-z

**Published:** 2019-01-08

**Authors:** A. Vienne-Jumeau, F. Quijoux, P. P. Vidal, D. Ricard

**Affiliations:** 1 Cognition and Action Group, Cognac-G, CNRS UMR 8257, Université Paris Descartes, Sorbonne Paris Cité, Service de Santé des Armées, 45 rue des Saints Pères, 75006 Paris, France; 2 ORPEA Group, 12 rue Jean Jaurès, CS 10032, 92813 Puteaux Cedex, France; 3 Institute of Information and Control, Hangzhou Dianzi University, Zhejiang, 310018 China; 4 Service de Neurologie de l’Hôpital d’Instruction des Armées de Percy, Service de Santé des Armées, 101 avenue Henri Barbusse, 92140 Clamart, France; 5 Ecole du Val-de-Grâce, Ecole de Santé des Armées, 1 Place Alphonse Laveran, 75005 Paris, France

**Keywords:** Multiple sclerosis, Gait analysis, Gait quantification, Gait disorders, Wearable inertial sensors, Inertial measurement unit, Accelerometer

## Abstract

**Background:**

Gait impairment is a hallmark of multiple sclerosis (MS) which significantly endangers the quality of life of the individual. Inertial measurement units (IMUs) are small, light wearable sensors that can be used in routine neurological practice. InertiaLocoGraphy (ILG), the quantification of gait with IMUs, has proven useful to detect early changes in MS undetectable with standard stopwatch-timed measures. Still, whether such markers are useful for evaluating the severity of the disease remains unknown. Therefore, the correlation between ILG and disease progression would be worth exploring.

**Methods:**

We will search MEDLINE via PubMed, Cochrane, and EMBASE electronic databases to identify articles published before May 2, 2018 that measure gait using IMUs in MS patients. In addition, grey literature will be searched. Inclusion criteria will be adults with a clinical diagnosis of MS and gait measured by using inertial sensors. We will exclude from the meta-analysis articles that do not provide sufficient data for evaluating the correlations between ILG parameters and disease severity as measured by at least one of the six following tests: the Expanded Disability Status Scale (EDSS), the Multiple Sclerosis Walking Scale-12 (MSWS), the Multiple Sclerosis Severity Score (MSSS), the Multiple Sclerosis Impact Scale (MSIS-29), the Multiple Sclerosis Functional Composite (MSFC), and the Timed 25-ft Walk Test (T25FW). Extracted data from included articles will be presented descriptively, and effect sizes will be computed based on the recommendations from the Cochrane Collaboration handbook and RevMan software.

**Discussion:**

Identifying changes in disease state throughout the course of MS is essential for optimal care. Current clinical and performance tests allow for identifying advanced gait alteration but lack sensitivity to detect subtle gait change. IMUs can be easily used in clinical practice to quantify gait in MS patients. Nevertheless, whether these outcomes are clinically relevant is uncertain because no study has evaluated their correlation with disease severity across different settings. This systematic review and meta-analysis would bring insight into the potential of this outcome as a marker of disease evolution.

**Systematic review registration:**

This review was registered with the International Prospective Register of Systematic Reviews on May 2, 2018 (Registration: CRD42018092651). Both the search strategy and study protocol are available at https://www.crd.york.ac.uk/PROSPERO/display_record.php?RecordID=92651.

**Electronic supplementary material:**

The online version of this article (10.1186/s13643-018-0918-z) contains supplementary material, which is available to authorized users.

## Background

Multiple sclerosis is a heterogeneous demyelinating disease of the central nervous system with varying clinical presentation and progression. Gait impairment is a hallmark of MS and lower limb function is perceived as the most important bodily disability across the disease spectrum [1]. Thus, there is a need for objective gait assessment both in routine clinic care and in clinical research trials to improve gait and balance follow-up in people with MS. Mobility is measured by the Expanded Disability Status Scale (EDSS), which has been criticized for its lack of sensitivity to change [2, 3] and its high interrater variability [4]. The Multiple Sclerosis Severity Score (MSSS) is another scale, which is obtained by normalizing the EDSS score for disease duration [5]. Patient-reported outcomes are also useful to inform disease severity; the Multiple Sclerosis Walking Scale-12 (MSWS) is a 12-item measure of the impact of MS on walking while the Multiple Sclerosis Impact Scale (MSIS-29) is a 29-item measure of the impact of MS on day-to-day life in the past 2 weeks. However, both are subjective. Gait speed is measured by using stopwatch-timed tests such as the Timed 25-ft Walk Test (T25FW), which has been criticized for being highly variable [6, 7]. Finally, the Multiple Sclerosis Functional Composite (MSFC) is an instrument used more and more frequently which encompass three tests, including the T25FW, expressed as a single score along a continuous scale [8]. However, it is prone to practice effects [9] and variability [6, 7].

Inertial measurement units (IMUs) are small, light integrated systems that measure linear and angular motion usually with a triad of accelerometers and triad of gyroscopes, often associated with a magnetometer. Motion capture systems based on these wearable sensors have become widely used for the biomechanical analysis of human movement. InertiaLocoGraphy (ILG), the quantification of gait with IMUs, was first reported 70 years ago [10] and has now been implemented in a wide range of neurological and non-neurological diseases [11, 12]. It can be used both at the hospital, mainly for short tests, and at home for more long-term physiological gait assessment [13]. ILG has proven useful to detect early changes in MS undetectable with already-mentioned assessments [1]. Still, whether such markers are useful for evaluating the severity of the disease remains unknown. Therefore, the correlation between ILG and disease progression would be worth exploring.

This meta-analysis is aimed at evaluating the value of inertial gait parameters in assessing disease severity in MS patients. Six objectives will be evaluated as follows:

Primary outcome:

- Correlation of ILG with EDSS

Secondary outcomes:

- Correlation of ILG with MSSS
- Correlation of ILG with MSWS
- Correlation of ILG with MSIS-29
- Correlation of ILG with stopwatch-timed T25FW
- Correlation of ILG with MSFC

Scales for which data for at least one parameter cannot be retrieved for at least two articles from different authors will only be mentioned and commented on in the discussion of the paper.

From our preliminary literature search, we expect the assessment of fall risk to be highly variable across studies. If not, the correlation of gait features with the assessment of fall risk will be added as a seventh objective.

## Methods/design

The literature search and analysis will follow the Preferred Reporting Items for Systematic Reviews and Meta-Analyses (PRISMA) [14] and Meta-analysis of Observational Studies in Epidemiology (MOOSE) [15] guidelines.

### Search strategy

We will search MEDLINE via PubMed, Cochrane, and EMBASE electronic databases to identify articles published before May 2, 2018, that measure gait using inertial sensors in MS patients. In addition, grey literature will be searched in Google Scholar, Opengrey.eu, Greylit.org, WorldCat, World Health Organization Clinical Trials Search Portal, ClinicalTrials.gov, and the European Union Clinical Trials Register. All reference lists and bibliographies of included studies will be reviewed for relevant studies that could be missed by this electronic search.

The search will involve the population-specific keywords “multiple sclerosis” or “MS” with intervention-specific terms such as “gait” or “walk*” or “step*” and comparison-specific keywords “sensor” or “wearable” or “inertial” or “IMU” or “accelerometer” or “gyroscope” or “motion analysis”. No keywords for the Outcome component of the PICO framework will be added to increase the sensitivity of the search. The Boolean word AND will be used between the PICO components.

### Inclusion criteria

Randomized controlled trials (RCTs), non-randomized controlled trials, and observational studies will be eligible for inclusion. Inclusion criteria will be adults with a clinical diagnosis of MS and gait measured by using inertial sensors (accelerometer and/or gyroscope).

### Exclusion criteria

We will exclude articles of studies that do not include people with MS (population criterion), quantify other activities such as standing or running or that assess general physical activities using only step count or walking bout length (GAIT criterion), and use sensors other than IMUs (IMU criterion).

We will exclude from the meta-analysis articles that do not provide sufficient data for pooling results despite authors being contacted for missing information (see paragraph Data extraction and analysis). Data will be considered sufficient in one of the three following situations: correlation between gait parameters with EDSS, MSSS, MSWS, MSFC, MSIS-29, or T25FW reported or obtained from the main author; raw values of gait parameters and EDSS, MSSS, MSWS, MSFC, MSIS-29, or T25FW reported or obtained from the main author for every patient; and raw values of gait parameters reported or obtained from the main author for groups of patients, with groups drawn from their EDSS, MSSS, MSWS, MSFC, MSIS-29, or T25FW values respectively (REPORTING criterion).

We expect very few studies set up at home. Because protocols at home vary widely from protocols in clinical or laboratory settings, ambulatory studies should be analyzed separately. Consequently, ambulatory studies will be excluded from the analysis if we cannot find at least three studies from which a common effect size can be drawn.

### Review process

Potentially eligible studies will be screened for eligibility independently by two review authors (FQ and AV). The population criterion will be assessed on the basis of titles and abstracts of papers, and the other criteria will be assessed in the main text. We will import articles to Zotero, and all articles will be reviewed (title, abstract, and main text when needed) to discard those that do not meet the criteria. In case of discrepancies between reviewers, they will discuss until a consensus is reached. If no consensus is reached, a third author (DR) will arbitrate. Eligible papers will be assessed for risk of bias and data will be extracted.

### Risk of bias appraisal

A risk of bias assessment will be performed by using a 20-item quality checklist for longitudinal studies that we adapted from Hubble et al. [16] by altering one item and adding three more items to better fit the requirements of our aim (Additional file 2).

Each article will be assessed by two assessors (FQ and AV), with each assessor blinded to the score given by the other. Disagreements will be discussed and the rounded mean of both scores will be chosen as the final score if no agreement is found.

### Data extraction and analysis

#### Study review

Upon selecting articles for inclusion, references will be imported in Microsoft Excel for data extraction. One assessor (AV) will extract and collate information. Another assessor (FQ) will verify the extracted data from all included articles. We will extract the following data:

- Study characteristics: authors, title, year of publication, inclusion and exclusion criterion, sample size
- Population characteristics: sex, age, weight, height, body mass index, EDSS, MSSS, MSWS, MSIS-29, MSFC, T25FW fall assessment
- Gait evaluation conditions: environment (laboratory versus ambulatory), floor type and sequence of steps, speed, sensitization tactics (eyes open or close, single, or double task), aid use
- Sensor characteristics: position, brand, and sampling frequency;
- Gait features: description and raw data when available
- Statistical analysis: test for normality, test used, whether correction for multiple comparison was applied, confounding factors, parameter significance

The data extraction tables will be pilot-tested and refined before extraction.

Correlation coefficients will be extracted when available. If correlation coefficients are not reported, the full raw data will be sought and retrieved. If neither correlation coefficients nor raw data are available, either will be requested from the main author (see paragraph Data extraction and analysis). If the main author cannot provide this information, the difference in means and the standardized mean difference will be retrieved.

When data are not available in the main text, we will search supplemental materials for more detailed information. When data on sensors (such as the sampling frequency) are not available even in supplemental materials, we will search for the data in preceding articles from the same author. For crossover studies, we will consider the data before the crossover started. Authors will be contacted up to three times via e-mail to obtain data not available in the main text. If data are only available by graphical representation, two authors (FQ, AV) will use Plot Digitizer to extract data from graphs: the final value will be the mean of these two extractions. Data extraction means will be reported in the manuscript.

#### Data pooling: parameter homogenization

First, to maximize the number of data points for a given parameter, we will try to derive from all studies any given parameter that was reported in at least one included study. For that aim, the following formulas will be implemented:

$\mathrm{CV}=\frac{\mathrm{SD}}{\mathrm{mean}}$ where CV is the coefficient of variation, SD is the standard deviation, mean is the mean of the same parameter;

$\mathrm{if}\ \mathrm{ri}=\frac{\left|P1-P2\right|}{\mathrm{mean}\left(P1,P2\right)}\ \mathrm{and}\ \mathrm{rf}=\frac{P1}{P2},\mathrm{then}:\mathrm{rf}=2\times \frac{\mathrm{ri}-1}{\mathrm{ri}+1}\ \mathrm{and}\ \mathrm{drf}=4\times \frac{\mathrm{dri}}{{\left(\mathrm{ri}+1\right)}^2}$ where ri and rf will both be use as an index of symmetry;

$$ \mathrm{swing}\ \mathrm{time}=\mathrm{step}\ \mathrm{time}-\mathrm{stance}\ \mathrm{time} $$

$$ \mathrm{double}\ \mathrm{stance}\ \mathrm{time}=\mathrm{stride}\ \mathrm{time}-2\times \mathrm{swing}\ \mathrm{time} $$

To minimize redundancy, when two proportional parameters are reported (e.g., step time or stride time likely appearing depending on the article), the parameter that was computed the least often in the included articles will be transformed by multiplying by the proportionality coefficient to match the other parameter (in the case above, the proportionality coefficient would be 1/2 if the stride time was the parameter the less computed between the two).

#### Data pooling: metrics

Second, we expect studies to assess disease severity using different metrics, mainly, correlation coefficients, between-group comparisons for two groups (standardized or unstandardized mean differences), between-group comparisons for several groups (eta squared, partial eta squared, and omega squared), or odds ratios. To conduct the meta-analysis across all of the effect sizes, these must be transformed into a common metric. Because thresholds for subgroups are not consensual, we will use the correlation coefficient as the effect size. We will transform the correlation coefficient (*r*) by using the Fisher *z* transformation (using *z* = atanh(*r*)) and perform the analysis using this index. This will allow us to use tests for normal distributions. Then, the summary values will be converted back to correlations for presentation (*r* = tanh(*z*)) [17].

#### Strategy for data synthesis

Extracted data from included articles will be presented descriptively, and effect sizes will be computed based on the recommendations from the Cochrane Collaboration handbook and RevMan software.

RevMan 5.3 will be used to create the forest plots for the meta-analysis. A fixed-effects model will be chosen when heterogeneity is low to moderate (*I*^2^ < 50%) [18]; otherwise, a random-effects model will be used.

#### Sensitivity analysis

When a given feature is reported in at least three studies from different authors, funnel plots will be used to search for possible publication bias. Sensitivity analysis will be used to explore the impact of recording settings (floor type [ground vs treadmill], sequence of steps [ambulatory vs clinical setting and U-turn vs no U-turn], speed instruction [convenient vs fastest]) on the computed gait features and their correlation with disease severity. The overall quality of the evidence for each outcome will be evaluated by using the GRADE criteria following the Cochrane Collaboration recommendations if enough RCTs and interventional studies are included [19].

## Discussion

Identifying changes in disease state throughout the course of MS is essential for optimal care [20]. Current clinical and performance tests (EDSS, MSSS, MSWS, MSIS-29, MSFC, T25FW) allow for identifying advanced gait alteration but lack sensitivity to detect subtle gait dysfunction or progression. IMUs can be easily used in clinical practice to quantify gait in MS patients. Nevertheless, whether these outcomes are clinically relevant is uncertain because no study has evaluated their correlation with disease severity across different settings. This systematic review and meta-analysis would bring insight into the potential of this outcome as a marker of disease evolution.

We acknowledge several limitations to this study. Although PRISMA guidelines will be adhered to and the methodology will be strictly followed, completely accounting for the limitations of included studies is impossible. Because MS patients present variable symptoms, populations will vary across studies. A recent meta-analysis evaluating gait alteration in MS revealed that gait studies included mainly individuals with low EDSS levels, which may limit the sensitivity as well as the external validity of the findings [21]. Interventions are also susceptible to heterogeneity because gait analysis protocols as well as algorithms used and features computed are numerous [15]. Because speed has been shown to depend on the set-up (time or length of the exercise) [13, 22], there might be significant heterogeneity for gait feature outcomes across studies. These elements can decrease the validity of the meta-analysis. Nevertheless, these weaknesses will be assessed to allow us to discuss results accordingly. Furthermore, grey literature will be searched in addition to traditional databases of published literature to try to limit publication bias. Another limitation lies in the fact that we will not review correlations of gait features to disease severity for various evolution states in a given patient. Therefore, we cannot directly conclude that a parameter that would prove to be associated with clinical scales in this review can be used as a severity marker in a given patient. Such assumption would require postulating that individual-based correlations can be interpolated from population-based correlations, which must be verified. For this aim, longitudinal studies only should be included, which we expect to be impossible at this time because of their small number in the literature. Furthermore, the quantitative synthesis of the effect of sensitizing conditions—for example, fatigued, dual-tasking, eyes-closed walk, narrow-step width, and obstacle negotiation—on altered gait parameters identified by this review should also be investigated to enhance the assessment and treatment of gait in individuals with MS.

## Additional files

Additional file 1: Standardized form for data extraction. (DOCX 40 kb)

Additional file 2: 20-item quality checklist. (DOCX 42 kb)

## Abbreviations

EDSS: Expanded Disability Status Scale
ILG: InertiaLocoGraphy
IMU: Inertial measurement unit
MS: Multiple sclerosis
MSFC: Multiple Sclerosis Functional Composite
MSIS-29: Multiple Sclerosis Impact Scale
MSSS: Multiple Sclerosis Severity Score
MSWS: Multiple Sclerosis Walking Scale-12
T25FW: Timed 25-ft Foot Walk
