# Supplementary material for: Value of gait analysis for measuring disease severity using inertial sensors in patients with multiple sclerosis: protocol for a systematic review and meta-analysis
Source: Syst Rev. 2019 Jan 8;8:15. doi: 10.1186/s13643-018-0918-z (PMC6325868; doi:10.1186/s13643-018-0918-z)
Supplement: Supplementary file 1 — Standardized form for data extraction. (DOCX 40 kb) [file 13643_2018_918_MOESM1_ESM.docx]

# Additional file 1: Standardized form for data extraction

| Theme | Data | Unavailable |
| --- | --- | --- |
| **Study characteristics** | | |
| authors |  | ☐ |
| title |  | ☐ |
| year of publication |  | ☐ |
| study type | ☐ interventional study: ☐ RCT ☐ non RCT  ☐ observational longitudinal study  ☐ observational transversal study |  |
| inclusion criteria | ☐ adults with a diagnosis of MS  ☐ inertial sensors used for gait analysis | ☐ |
| exclusion criteria | standing ☐  running ☐  general physical activities ☐  other sensors than IMUs ☐  do not include MS people ☐ | ☐ |
| sample size | HS:  MS: | ☐ |
| **Population characteristics** | | |
| participants’ gender | HS: Male: Female:  MS: Male: Female: | ☐  ☐ |
| age | HS:  MS: | ☐  ☐ |
| weight | HS:  MS: | ☐ |
| height | HS:  MS: | ☐ |
| BMI | HS:  MS: (gp1: gp2: gp3: gp4: ) | ☐ |
| EDSS | MS: (gp1: gp2: gp3: gp4: ) | ☐ |
| FIS or MFIS | MS: (gp1: gp2: gp3: gp4: ) | ☐ |
| MSWS | MS: (gp1: gp2: gp3: gp4: ) | ☐ |
| fall assessment | method: ☐ number of falls in _____ months  ☐ fall risk score  ☐ other  HS:  MS: (gp1: gp2: gp3: gp4: ) | ☐ |
| gait evaluation condition |  | ☐ |
| environment | ☐ laboratory  ☐ ambulatory | ☐ |
| floor type | ☐ ground  ☐ treadmill | ☐ |
| sequence of steps | ☐ U-turn  ☐ no U-turn | ☐ |
| speed | ☐ convenient  ☐ fastest  ☐ population based average speed  ☐ other imposed | ☐ |
| sensitization tactics | ☐ eyes open  ☐ eyes closed  ☐ single task  ☐ double task | ☐ |
| **Sensor characteristics** | | |
| position |  | ☐ |
| brand |  | ☐ |
| sampling frequency |  | ☐ |
| **Gait features** | | |
| description |  | ☐ |
| full raw data available | ☐ yes  ☐ no | ☐ |
| extracted data | ☐ Pearson correlation coefficients available  ☐ Spearman correlation coefficients available  ☐ Eta squared coefficients available  ☐ correlation coefficients computed from group differences in means and standardized mean difference  ☐ correlation coefficients computed from full raw data  ☐ data extracted from graphical representation | ☐ |
| **Statistical analysis** | | |
| test for normality |  | ☐ |
| tests used |  | ☐ |
| correction for multiple comparison |  | ☐ |
| confounding factors |  | ☐ |
| parameter significance |  | ☐ |
| Possible inclusion for meta-analysis | ☐ yes, without the authors’ data transmission  ☐ yes, with the authors’ data transmission  no, do not provide sufficient data ☐  no, do not provide correlation coefficient ☐  no, do not provide raw values of gait parameters ☐ | |
