# Supplementary material for: Value of gait analysis for measuring disease severity using inertial sensors in patients with multiple sclerosis: protocol for a systematic review and meta-analysis
Source: Syst Rev. 2019 Jan 8;8:15. doi: 10.1186/s13643-018-0918-z (PMC6325868; doi:10.1186/s13643-018-0918-z)
Supplement: Supplementary file 2 — 20-item quality checklist. (DOCX 42 kb) [file 13643_2018_918_MOESM2_ESM.docx]

# Additional file 2: 20-item quality checklist

**Reporting**

1. Is the hypothesis/aim/objective of the study clearly described? (1 point)
2. Are the main outcomes to be measured clearly described in the Introduction or Methods section? (1 point)

If the main outcomes are first mentioned in the Results, the score should be 0.

3. Is the protocol clearly described? (2 points)

The description should include the eight following elements: ambulatory or distance or time of walk, type of floor (ambulatory, treadmill, unlevelled, slop), sequence of steps (ambulatory or U-turn), sequence of steps (ambulatory or sit-to-stand transition), sensor type, sensor frequency, sensor position, and speed (self-selected or imposed). Give 0 points if two or more elements are missing, 1 point if one element is missing, 2 points if the eight elements are specified.

1. Are walking bout detection, walking segment detection and step detection methods clearly described or referred to? (1 point)

Walk detection refers to detection of walking bouts (required for ambulatory studies or when parameters are computed on the whole signal, but the whole time of walk is included in the processing). Segment detection includes detection of transition (acceleration and deceleration phases and U-turn when not included in the analyzed steps). Step detection includes detection of all steps. Description should allow for answering the following three questions: Is the method automatic or manual? Which signal is analyzed (IMUs or other sensor)? If it relies on only IMUs, which axis and which peak is used? When needed, the three elements should be explained in the methods or refer to an article with an explanation that allows for answers to the above questions. Give 0 point if two or more elements are not well described, 1/2 point if one element is not well described, 1 point if all elements are well described. If neither of the three elements are needed (eg, laboratory gait analysis with first 5 and last 5 sec discarded and parameters are computed on the whole signal), the item is not quoted.

1. Are the number of trials and steps included in the analysis clearly specified? (1 point)
2. Are the characteristics of the patients included in the study clearly described? (1 point)

In cohort studies and trials, the inclusion and/or exclusion criteria should be given. In case–control studies, a case-definition and the source for controls should be given.

1. Are the distributions of principal confounders in each group of subjects to be compared clearly described? (2 points)

A list should be given.

8. Are the main findings of the study clearly described? (1 point)

When they exist, quantitative findings should be reported in the Results section and discussed in the Discussion section for the main findings.

1. Does the study provide estimates of the random variability in the data for the main outcomes? (1 point)

For non-normally distributed data, the interquartile range should be reported. For normally distributed data, the standard error, standard deviation or confidence intervals should be reported. If the distribution of the data is not described, it must be assumed that the estimates used were appropriate and the item should be scored 1.

1. Have actual probability values been reported (eg, 0.035 rather than <0.05) for the main outcomes (when significant) except where the probability value is less than 0.001%? (1 point)

When no result is significant, the item is not quoted.

**External validity**

1. Were the subjects asked to participate in the study representative of the entire population from which they were recruited? (1 point)

The study must identify the source population for patients and describe how the patients were selected. Patients will be representative if they comprised the entire source population, an unselected sample of consecutive patients, or a random sample. Random sampling is only feasible when a list of all members of the relevant population exists. When a study does not report the proportion of the source populations from which the patients are derived, the score should be 0 (unable to determine).

1. Were those subjects who were prepared to participate representative of the entire population from which they were recruited? (2 points)

The proportion of those asked who agreed should be stated. Validation that the sample was representative would include demonstrating that the distribution of the main confounding factors was the same in the study sample and the source population.

13. Was there validation of the sensor used? (1 point)

If the sensor is self-made and no analysis of reliability was evident in the article or a previous article, the score should be 0.

**Internal validity - Bias**

1. If any of the results of the study were based on “data dredging”, was this made clear? (1 point)

Any analyses that were not planned at the outset of the study should be clearly indicated.

If no retrospective unplanned subgroup analyses were reported, then score 1.

15. Were the statistical tests used to assess the main outcomes appropriate? (1 point)

If no test for normality or no post-hoc corrections was performed when needed, only 1/2 point should be given. If both criteria fail, score should be 0.

16. Were the main outcome measures used accurate (valid and reliable)? (1 point)

For studies for which the outcome measures are clearly described, the score should be 1. For studies that refer to other work or demonstrate that the outcome measures are accurate, the score should be 1.

**Internal validity - Confounding (Selection Bias)**

1. Were the patients in different intervention groups (trials and cohort studies) or were the cases and controls (case-control studies) recruited from the same population? (1 point)

Patients for all comparison groups should be selected from the same hospital(s) or institution(s). Patients and controls should not have significantly diﬀerent ages. No point should be given when no information can be found regarding the source of patients included.

1. Were study subjects in different intervention groups (trials and cohort studies) or were the cases and controls (case-control studies) recruited over the same period of time? (1 point)
2. Was there adequate adjustment for confounding in the analyses from which the main findings were drawn? (1 point)

This question should be scored 0 for trials if the main conclusions of the study were 1) based on analyses of treatment rather than intention to treat; 2) the distribution of known confounders in the diﬀerent treatment groups was not described; or 3) the distribution of known confounders differed between the treatment groups but was not taken into account in the analyses. For non-randomised studies, if the eﬀect of the main confounders was not investigated or confounding was demonstrated but no adjustment was made in the final analyses, the question should be scored 0.

**Power**

1. Did the study have sufficient power to detect a clinically important effect if the probability for a difference due to chance is less than 5%? (5 points)

|  | Size of smallest group | Power estimate | Score |
| --- | --- | --- | --- |
| A | <n1 | 70% | 0 |
| B | n1-n2 | 80% | 1 |
| C | n3-n4 | 85% | 2 |
| D | n5-n6 | 90% | 3 |
| E | n7-n8 | 95% | 4 |
| F | n+ | 99% | 5 |
